# Calcitonin gene-related peptide alters the firing rates of hypothalamic temperature sensitive and insensitive neurons

**DOI:** 10.1186/1471-2202-9-64

**Published:** 2008-07-11

**Authors:** Daniel C Braasch, Erin M Deegan, Eleanor R Grimm, John D Griffin

**Affiliations:** 1 Department of Biology and Program in Neuroscience, College of William and Mary, Williamsburg, Virginia 23187, USA

## Abstract

**Background:**

Transient hyperthermic shifts in body temperature have been linked to the endogenous hormone calcitonin gene-related peptide (CGRP), which can increase sympathetic activation and metabolic heat production. Recent studies have demonstrated that these centrally mediated responses may result from CGRP dependent changes in the activity of thermoregulatory neurons in the preoptic and anterior regions of the hypothalamus (POAH).

**Results:**

Using a tissue slice preparation, we recorded the single-unit activity of POAH neurons from the adult male rat, in response to temperature and CGRP (10 μM). Based on the slope of firing rate as a function of temperature, neurons were classified as either warm sensitive or temperature insensitive. All warm sensitive neurons responded to CGRP with a significant decrease in firing rate. While CGRP did not alter the firing rates of some temperature insensitive neurons, responsive neurons showed an increase in firing rate.

**Conclusion:**

With respect to current models of thermoregulatory control, these CGRP dependent changes in firing rate would result in hyperthermia. This suggests that both warm sensitive and temperature insensitive neurons in the POAH may play a role in producing this hyperthermic shift in temperature.

## Background

The preoptic and anterior regions of the hypothalamus (POAH) have been shown to be thermoregulatory control centers of the brain for integrating and responding to changes in peripheral, core, and hypothalamic temperature. Although stimulation of specific sites in the brainstem may selectively activate thermoregulatory responses, local warming of the POAH results in a general heat loss, while cooling initiates heat production [1]. Within this region, *in vivo *and *in vitro *electrophysiologic studies have identified thermally classified populations of neurons. The majority of these neurons are considered temperature insensitive, showing little or no temperature dependent changes in firing rate. However, approximately 30% of the neurons in the POAH can be classified as warm sensitive [2]. The firing rates of these neurons can be selectively increased with warming of the POAH or decreased with cooling of this region. In addition, warm sensitive neurons are responsive to variations in skin and spinal temperature, and their activity can be directly correlated with the activation of physiologic and behavioral thermoregulatory responses [3]. Therefore, warm sensitive neurons in the POAH appear to function as integrators of both central and peripheral thermal information, producing an efferent output that is directly responsible for the maintenance of body temperature. While both warm sensitive and temperature insensitive neurons have been shown to respond to specific endogenous mediators, there are no studies to date concerning the effects of the hormone CGRP on the activity of these neurons.

An enduring model of thermoregulatory control suggests that the activity of temperature insensitive neurons is integrated with the activity of warm sensitive neurons to determine a "set-point" temperature [4]. While the concept of a single "set-point" for all thermoregulatory effector responses has recently been challenged [5], this model does provide a testable basis for understanding how endogenous modulators like CGRP can alter thermoregulatory control. For example, it is clear that during fever, a hyperthermic shift in thermoregulation occurs, resulting in an increase in heat production until the new balance is reached at a higher core temperature. Evidence suggests that this hyperthermic shift results from the local production of prostaglandin E_2 _(PGE_2_) and its effects on the activity of specific populations of POAH neurons [6]. This has been demonstrated in a recent study which found that PGE_2 _directly inhibits the firing rates of most warm sensitive neurons and increases the activity of some temperature insensitive neurons in the most anterior regions of the POAH [7]. Based on the model, either of these responses would result in hyperthermia and would predict that other mediators of hyperthermia, like CGRP, would have similar effects.

While fever is characterized by a long term hyperthermic shift in thermoregulatory control, the hyperthermia generated by CGRP can be much shorter in duration and results from both an increase in sympathetic activation and metabolic rate [8,9]. This response is a common complication of anti-androgen treatment for prostate cancer in men that can be correlated with a rise in the level of the hormone CGRP [10]. Experimentally, several studies have demonstrated similar increases in sympathetic activation and skin temperature when CGRP is elevated through intravenous injection [8,9,11]. Additionally, injection of CGRP directly into the dorsal medial hypothalamus or the ventral medial hypothalamus, two effector regions involved in thermoregulatory control, results in an increase in oxygen consumption, heart rate and core temperature in rats [8]. Therefore, CGRP may act on the central nervous system (CNS) to produce a hyperthermic shift in thermoregulatory control.

Anatomical evidence also supports a central role for CGRP in regulating body temperature. It has been identified in the somas of neurons located in several nuclei of the hypothalamus [12], and the levels of CGRP mRNA expression within the POAH have been shown to increase after castration of male rats and ovarianectomization of female rats [13]. In addition, distinct binding sites for this endogenous mediator are distributed throughout the rodent brain, including the POAH [14], and CGRP application in this region stimulates a rapid increase in CGRP receptors [15].

The mechanism by which CGRP can stimulate a hyperthermic shift in body temperature is unknown. We hypothesize that like PGE_2_, which mediates the rise in temperature that is a fever and accompanies stimulation of the immune system, CGRP stimulates this response through its selective effects on the firing rates of warm sensitive and temperature insensitive neurons in the POAH. Based on the proposed functional role of these neurons in thermoregulatory networks, these distinct responses to CRGP could lead to a transient hyperthermic shift in thermoregulatory control.

## Results

The firing rates of forty-five POAH neurons were recorded and characterized for responses to temperature and the endogenous hormone CGRP. As mentioned in the Methods, the thermosensitivity (impulses^.^s^-1.^°C^-1^) of each neuron was determined by plotting firing rate as a function of temperature. For neurons that were classified as warm sensitive, the regression coefficient (m) of this plot was at least 0.8 impulses^.^s^-1.^°C^-1^. All other neurons were classified as temperature insensitive. Thirty-eight neurons showed little change in firing rate in response to variations in temperature and were classified as temperature insensitive (m = 0.19 ± 0.03 impulses^.^s^-1.^°C^-1^). The remaining seven neurons showed increases in firing rate in response to changes in temperature and were classified as warm sensitive (m = 1.2 ± 0.32 impulses^.^s^-1.^°C^-1^).

**Figure 1:**
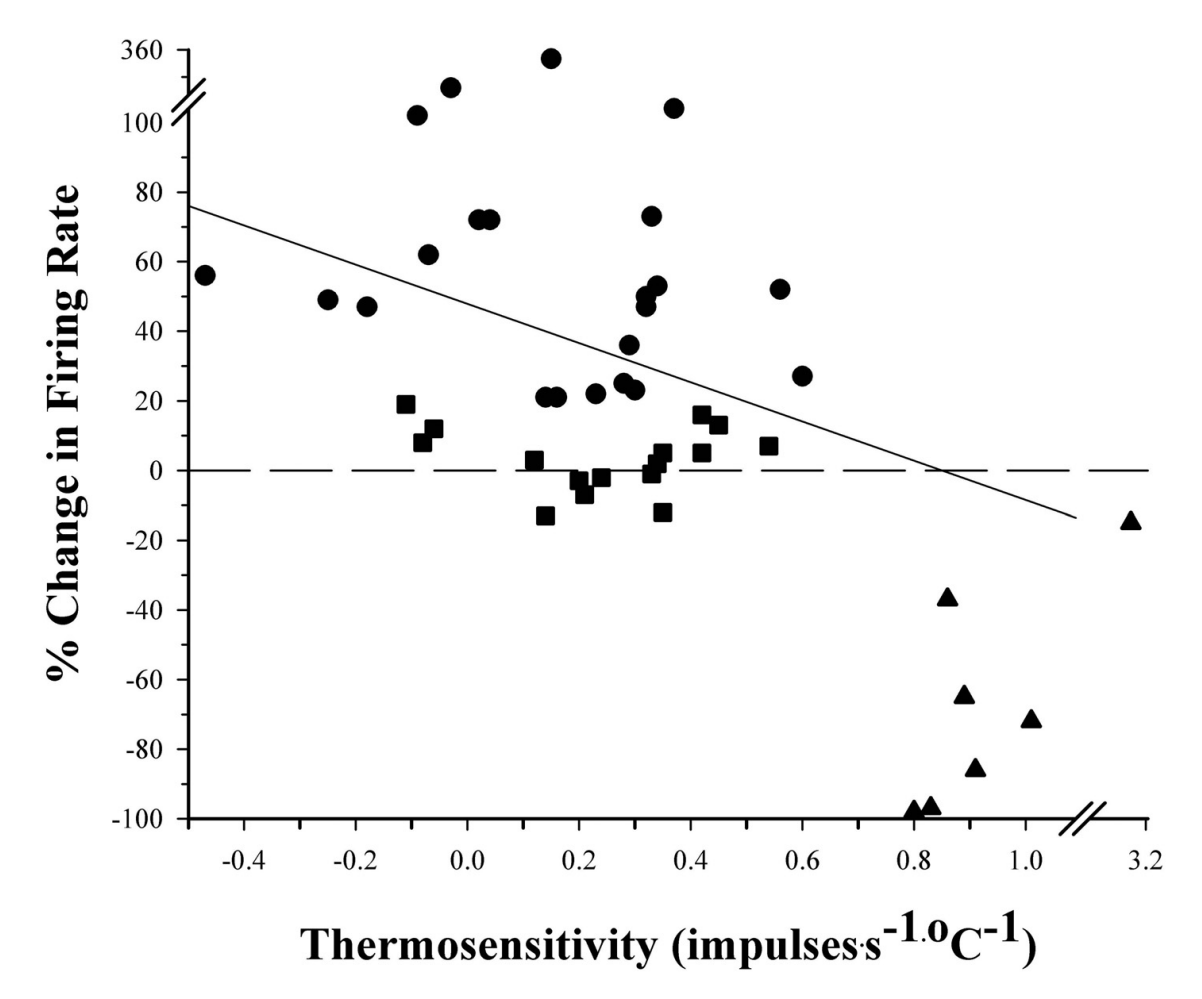
**The effect of CGRP on the firing rates of POAH neurons**. The percent change in firing rate in response to CGRP for each neuron is plotted as a function of thermosensitivity (impulses^.^s^-1.^°C^-1^). The dashed line indicates 0% change in firing rate. A regression fit to the data is indicated by the solid diagonal line (r = 0.14). Warm sensitive neurons (m ≥ 0.8 impulses^.^s^-1.^°C^-1^) all showed a significant decrease in firing rate in response to CGRP and are represented by triangles. Temperature insensitive neurons (m < 0.8 impulses^.^s^-1.^°C^-1^), either did not response to CGRP (squares) or increased their firing rates (circles).

**Figure 2:**
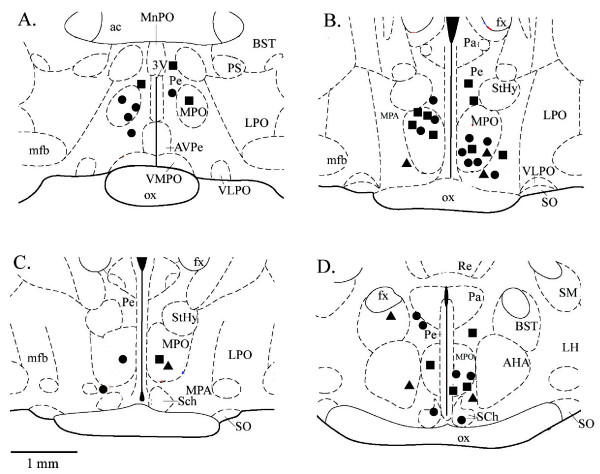
**Recording locations of POAH neurons in response to temperature and CGRP**. Diagrams of the POAH are shown in the coronal plane, with the most rostral in the upper left, progressing caudally in clockwise order. Distance from bregma: A = -0.3 mm; B = -0.8 mm; C = -0.92 mm; D = -1.4 mm. Diagrams were adapted from an atlas of the rat brain (Paxinos and Watson, 1998). Circles: insensitive neurons which showed a significant increase in firing rate. Squares: insensitive neurons that did not show a change in firing rate. Triangles: warm sensitive neurons which showed a significant decrease in firing rate. 3 V, third ventricle; ac, anterior commissure; AHA, anterior hypothalamic area; AVPe, anteroventral periventricular nucleus; BST, bed nucleus stria terminalis; fx, fornix; LH, lateral hypothalamus; LPO, lateral preoptic area; MnPO, median preoptic nucleus; MPO, medial preoptic nucleus; MPA, medial preoptic area; mfb, median forebrain bundle; ox, optic chiasm; Pa, paraventricular nucleus; Pe periventricular nucleus; PS, parastrial nucleus; Re, reunions thalamic nucleus; Sch, suprachiasmatic nuceus; SM, stria medullaris of thalamus; SO, supraoptic nucleus; StHy, striohypothalamic nuc.; VLPO, ventrolateral preoptic area; VMPO, ventromedial preoptic area.

Once thermosensitivity was determined, each neuron's firing rate was recorded during exposure to CGRP. A change in firing rate in response to CGRP was determined by statistical significance (See Methods; P ≤ 0.05). In response to CGRP, the majority of temperature insensitive neurons (n = 22) showed an increase in firing rate from baseline, increasing by an average of 47% (Fig. 1). The mean firing rate of these CGRP responsive temperature insensitive neurons increased from 3.5 impulses^.^s^-1 ^to 5.1 impulses^.^s^-1^, returning to a mean of 3.6 impulses^.^s^-1 ^during the washout period (Table 1). As shown in Figure 1, all of the warm sensitive neurons responded to CGRP with a decrease in firing rate, with an average change in firing rate of -59%. The mean firing rate of these warm sensitive neurons decreased from 8.2 impulses^.^s^-1 ^to 3.3 impulses^.^s^-1 ^in response to CGRP and returned to 8.1 impulses^.^s^-1 ^during washout. With respect to CGRP, responsive neurons were found in several hypothalamic nuclei, with the majority located within or in close proximity to the medial preoptic nucleus (Fig. 2).

**Table 1:** The effects of CGRP on the firing rates of anterior hypothalamic neurons

			Firing Rate (impulses^.^s^-1 ^± S.E.)		
			
Thermosensitivity (impulses^.^s^-1.^°C^-1^)	Response	N	Baseline	CGRP	Washout
Insensitive (< 0.8)					
	No Change	16	4.2 ± 0.3	4.4 ± 0.3	4.1 ± 0.6
	Increase	22	3.5 ± 0.5	5.1 ± 0.6*	3.6 ± 0.4
Warm (≥ 0.8)					
	Decrease	7	8.2 ± 0.8	3.3 ± 0.6*	8.1 ± 0.5

*Significantly different from Baseline firing rate (Paired T-test p < 0.05)

**Figure 3:**
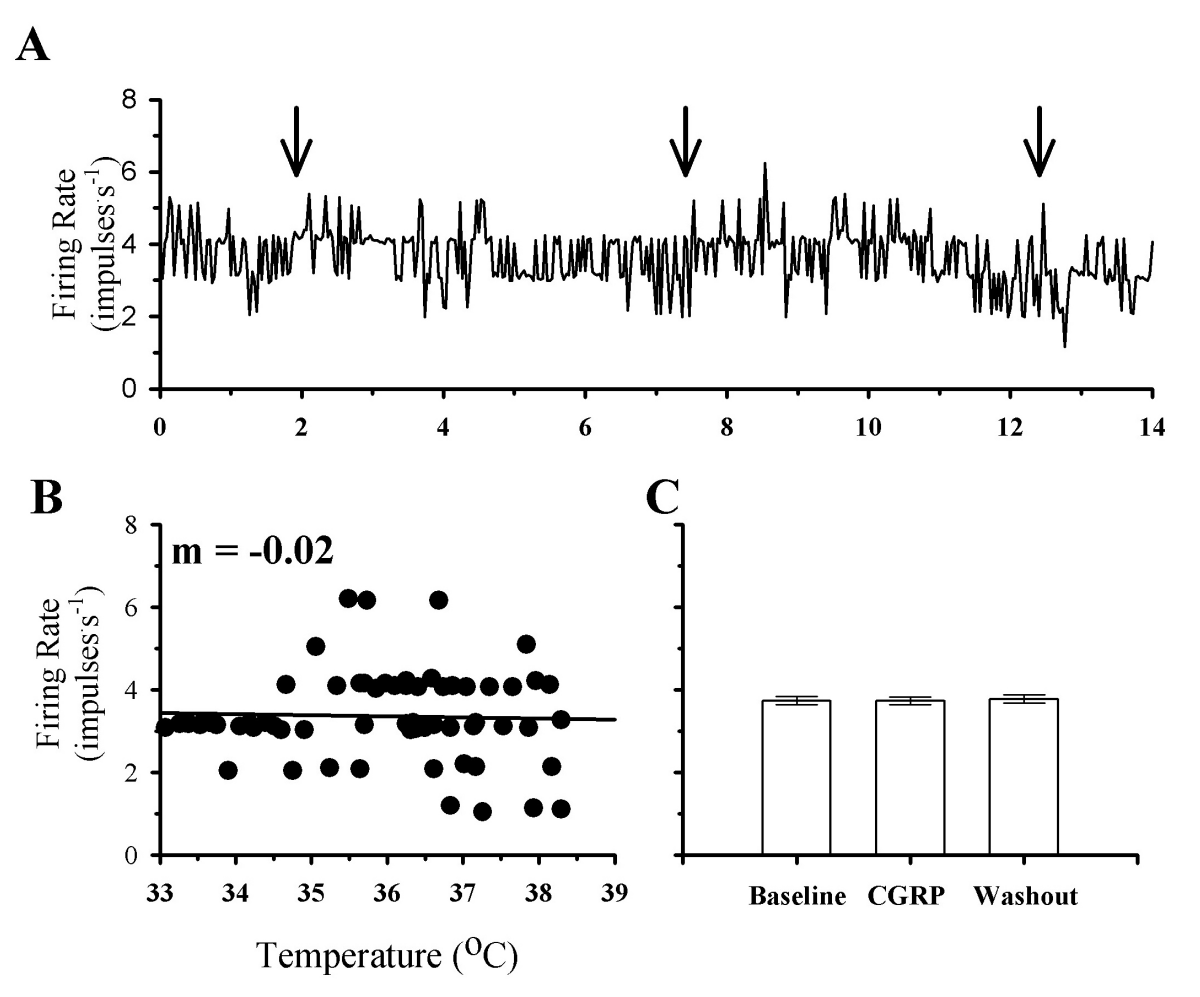
**The effect of CGRP on the firing rate of a non-responsive temperature insensitive neuron**. **A**, shows the firing rate of this neuron during two consecutive exposures to CGRP. The beginning of a CGRP exposure is indicated by the arrows. In **B**, firing rate is plotted as a function of temperature. In **C**, the average firing rates during the first exposure to CGRP are plotted for one minute segments of activity during Baseline (3.7 ± 0.1 impulses^.^s^-1^), CGRP (3.7 ± 0.3 impulses^.^s^-1^), and Washout (3.8 ± 0.1 impulses^.^s^-1^). For the bar graphs in C, standard error bars may be difficult to visualize.

While CGRP increased the firing rates of the majority of POAH temperature insensitive neurons, some (n = 16) did not respond to CGRP. Figure 3 shows the firing rate of a non-responsive temperature insensitive neuron. During a variation in temperature, firing rate showed little change (Fig. 3B). In addition, firing rate did not change in response to CGRP (Fig. 3A &3C). Repeat exposure to CGRP also had no effect on firing rate (Figure 3A).

**Figure 4:**
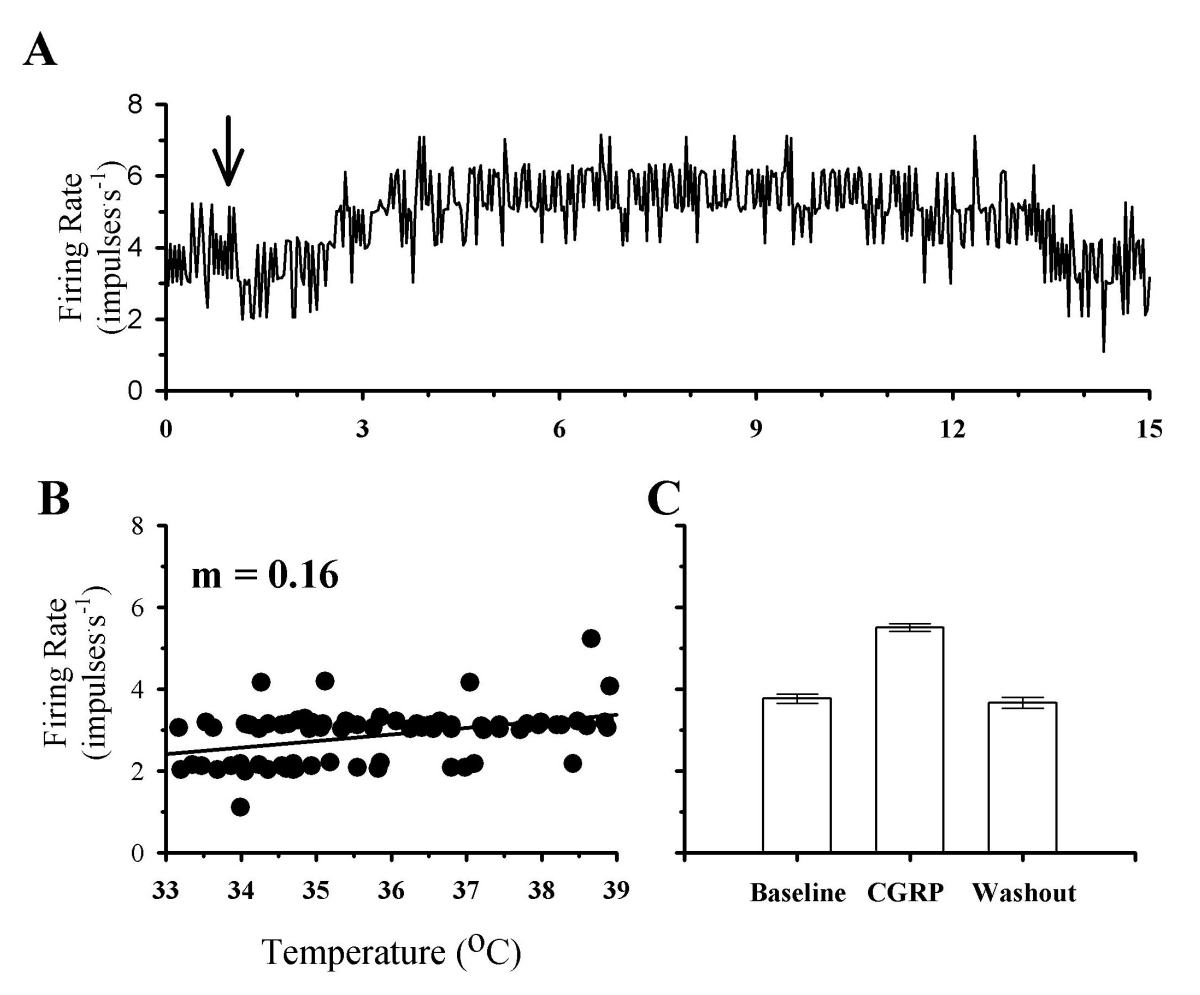
**The effects of CGRP on the firing rate of a responsive temperature insensitive neuron**. **A**, shows the firing rate of this neuron in response to CGRP. The beginning of a CGRP exposure is indicated by the arrow. In **B**, firing rate is plotted as a function of temperature. In C, the average firing rates are plotted for one minute segments of activity during Baseline (3.7 ± 0.1 impulses^.^s^-1^), CGRP (5.6 ± 0.1 impulses^.^s^-1^), and Washout (3.7 ± 0.1 impulses^.^s^-1^). For the bar graphs in C, standard error bars may be difficult to visualize.

Fig. 4 shows the firing rate of a temperature insensitive neuron that did change firing rate during exposure to CGRP. In response to a variation in temperature, this neuron showed little change in firing rate (Fig. 4B). However, after exposure to CGRP, firing rate increased by 47% and remained at this elevated level for approximately 10 minutes (Fig. 4A &4C).

**Figure 5:**
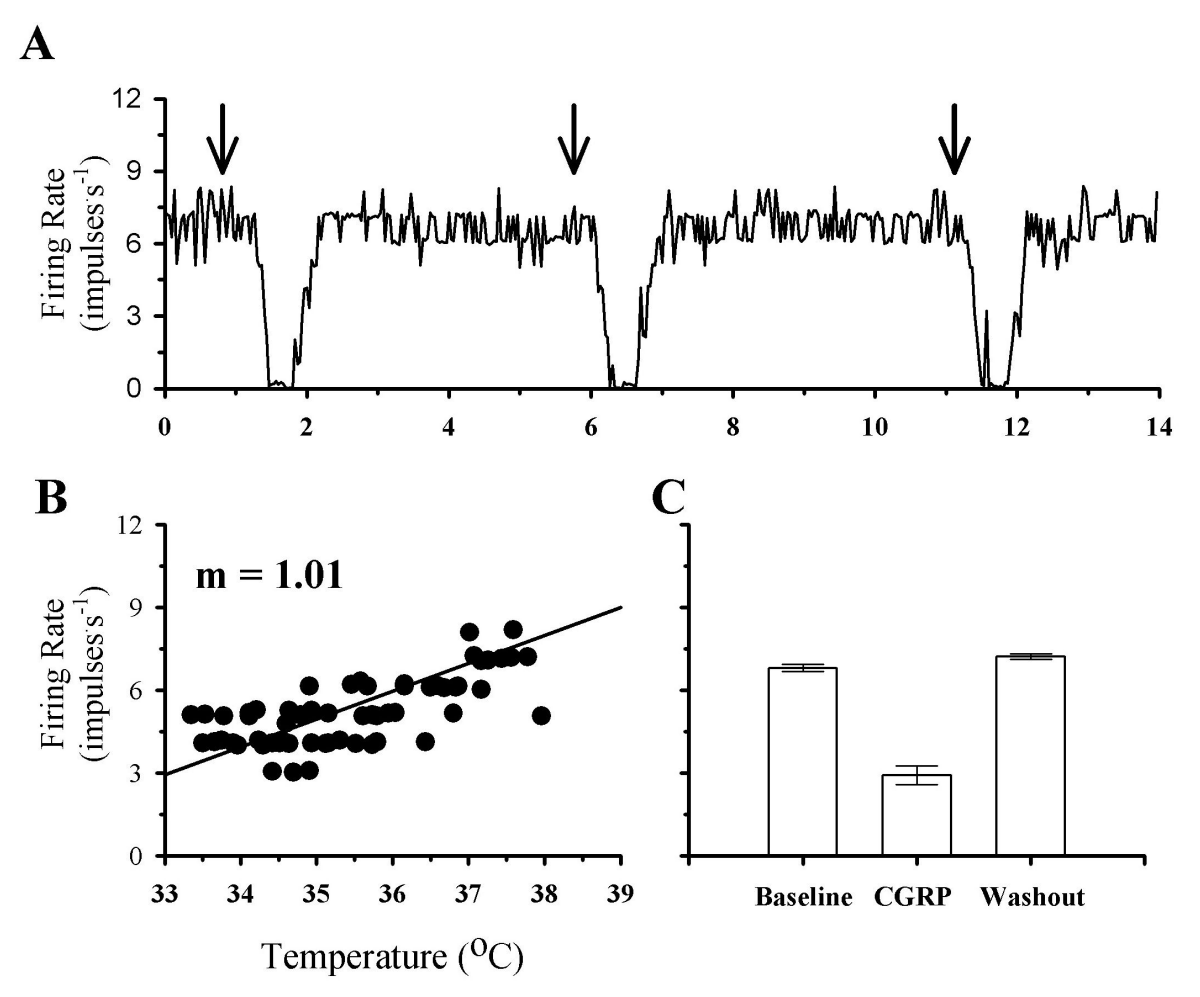
**The effects of CGRP on the firing rate of a warm sensitive neuron**. **A**, shows the firing rate of this neuron during three consecutive exposures to CGRP. The beginning of a CGRP exposure is indicated by the arrows. In **B**, firing rate is plotted as a function of temperature. In **C**, the average firing rates during the first exposure to CGRP are plotted for one minute segments of activity during Baseline (6.8 ± 0.1 impulses^.^s^-1^), CGRP (2.9 ± 0.3 impulses^.^s^-1^), and Washout (7.2 ± 0.1 impulses^.^s^-1^). For the bar graphs in C, standard error bars may be difficult to visualize.

In contrast to the changes in firing rate recorded from responsive temperature insensitive neurons, all of the warm sensitive neurons in this study showed decreases in firing rate in response to CGRP. Figure 5 shows the firing rate of a warm sensitive neuron. A change in temperature resulted in a correlated change in firing rate, with firing rate increasing during warming and decreasing with cooling (Fig. 5B). In response to CGRP, firing rate was significantly decreased for a short period of time, returning to baseline levels within a few minutes (Fig. 5A &5C). This response to CGRP was repeatable, as shown by the two additional exposures to CGRP, both resulting in a transient decrease in firing rate (Fig. 5A).

## Discussion

In response to increased levels of the endogenous hormone CGRP, body temperature has been shown to rise [8,9,11]. While the effects of CGRP include sympathetic activation and increased metabolic heat production [10], little is known about the neural activation that leads to these effector responses that cause hyperthermia. However, there is clear evidence for the presence of CGRP receptors in the POAH, suggesting that these responses are mediated through the effects of CGRP on neurons within this thermoregulatory control center of the brain [14].

The POAH has been shown to be at the top of a hierarchy of thermoregulatory control regions throughout the CNS. Within the POAH, the neuronal model first proposed by Hammel [4], suggests that warm sensitive and temperature insensitive neurons provide contrasting synaptic input to populations of effector neurons which control heat loss and heat production responses. When excitatory input from warm sensitive neurons falls below the level of inhibitory input from temperature insensitive neurons, effector neurons, which control heat loss, reduce their activation of physiologic and behavioral responses and, as a result, body temperature rises. In contrast, heat production is controlled by effector neurons which receive inhibitory synaptic input from warm sensitive neurons and excitation from temperature insensitive neurons. If inhibition from warm sensitive neurons decreases, it follows that these effector neurons increase their activity, stimulating an increase in heat production. Therefore, the CGRP-dependent decreases in the activity of warm sensitive neurons could likely result in hyperthermia due to an increase in heat production and decreased heat loss. Additionally, hyperthermia could also result from an increase in the firing rates of temperature insensitive neurons. Our results clearly show that CGRP can alter the activity of thermoregulatory neurons in the POAH, by decreasing the firing rates of warm sensitive neurons and increasing the firing rates of the majority of temperature insensitive neurons (Table 1).

All of the effects of CGRP are exerted at high affinity receptors that are distributed in a regionally specific manner in the CNS [14,16,17]. The functional binding site for CGRP has been shown to be a heterodimer [18] composed of a G-protein coupled receptor, the calcitonin receptor-like receptor [19], and a single transmembrane accessory protein, termed the receptor activity modifying protein-1 [20]. In the presence of CGRP, a stimulatory G-protein is activated, whose alpha-subunit mediates the activation of adenylyl cyclase [21]. While this process has been shown to alter the activity of several types of potassium conductance in smooth muscle and peripheral neurons, in cortical neurons, CGRP-dependent activation of AC has been shown to alter the activity of the potassium A-current (I_A_) [22]. Within the POAH, changes in the intracellular concentrations of cAMP and the activity of I_A _can regulate the neuronal firing rate and thermosensitivity [23,24]. Therefore, it may be speculated that these two CGRP-dependent mechanisms could play a role in producing the firing rate responses demonstrated in our current study. It is worth noting that PGE_2_, acting at EP_3 _receptors, activates AC and reduces the firing rates of warm sensitive neurons and thus, may be working to produce hyperthermia through a similar intracellular pathway [6,7].

With respect to our hypothesis that CGRP plays a role in mediating a transient hyperthermia, it is clear that CGRP is having a selective effect on the activity of POAH neurons that can be correlated with neuronal thermosensitivity (Figure 1). All of the warm sensitive neurons responded with a decrease in firing rate, and while temperature insensitive neurons either responded to CGRP with an increase in firing rate or did not respond, none of them showed a decrease in firing rate. (Table 1). Unlike PGE_2 _which mediates a thermogenic rise in core temperature during an immune response [6], CGRP responsive neurons were not restricted to any particular region or cell group within the POAH (Figure 2). While we do not yet know the functional role these neurons have in the neural pathways responsible for regulating specific thermoregulatory effector systems, we have clearly shown a selective effect for thermoregulatory neurons to CGRP that may lead to the initiation of an increase in body temperature.

## Conclusion

With respect to current models of thermoregulatory control, the results suggest that CGRP dependent changes in firing rate would result in hyperthermia. Either an increase in the firing rates of temperature insensitive neurons or a decrease in the firing rates of warm sensitive neurons would result in a shift in the thermoregulatory set point to a higher temperature.

Therefore, both warm sensitive and temperature insensitive neurons in the POAH may play a role in producing this hyperthermic shift in temperature.

## Methods

To record the extracellular single-unit activity of POAH neurons, tissue slices were prepared from male Sprague-Dawley rats (100–150 g) that were housed under standard conditions (23°C, 12:12-h light:dark cycle, with lights on at 8:00 am) and given food and water ad lib. Prior to each recording session, a rat was anesthetized with isofluorine and euthanized by quick decapitation, following procedures that have been approved by the University's Animal Care and Use Committee. After quick brain removal, a tissue block containing the POAH was cut and coronal slices (400 μM) were sectioned using a vibratome. The tissue slices were immediately transferred to an interface recording chamber. Throughout the recording session, the tissue slices were continuously perfused (1–2 ml^.^min^-1^) with an artificial cerebral spinal fluid (aCSF; 124 NaCl, 26 Na HCO_3_, 10 glucose, 5 KCl, 2.4 CaCl_2_, 1.3 MgSO_4_, and 1.24 KH_2_PO_4_), which was oxygenated (95% O_2 _- 5% CO_2_) and heated to an approximate temperature of 36°C. Tissue temperature was continuously monitored by a thermocouple which was placed in close proximity to the tissue slices.

Extracellular single-unit recordings were made using glass microelectrodes that had tip diameters of ~1 μm and were filled with 3 M NaCl. An Xcell-3 amplifier (FHC Inc.) was used to record electrical signals, which along with temperature, were recorded to digital tape for later analysis. The firing rate was determined over 1 second intervals, in which action potentials were isolated (signal to noise ratio of ≥ 3:1) and counted using a window discriminator and rate interval monitor (FHC Inc.). Once a stable level of activity was recorded for several minutes, the temperature was varied at least 2–3°C from the baseline temperature. By plotting firing rate as a function of temperature, the thermosensitivity (impulses^.^s^-1.^°C^-1^) was determined. In agreement with previous studies [25,26], warm sensitivity is defined as a regression coefficient (m) of at least 0.8 impulses^.^s^-1.^°C^-1^. Neurons that did not meet this minimum level of thermosensitivity were classified as temperature insensitive.

Once neuronal thermosensitivity was determined, and temperature was once again stable at ~36°C, recorded neurons were exposed to CGRP (Sigma Chemicals), which was diluted to a concentration of 10 μM in aCSF, warmed to 36°C, and oxygenated in a separate perfusion tube (pH 7.38). While firing rate was being monitored and during continuous perfusion with aCSF, a microdrop (~0.2 ml) of CGRP containing aCSF was applied directly to the surface of the tissue slice, in close proximity to the recording electrode [27,28]. Following exposure to CGRP, recordings were made for at least 10 minutes or until firing rate returned to baseline levels. After a recording session was completed, the firing rate of each neuron was digitized (60 Hz) for comparison and quantitative determination of responses to CGRP (Axoscope Software, Molecular Devices Inc.). One minute representative segments of firing rate activity were collected just prior to CGRP exposure (baseline), during the maximum response to CGRP, and after firing rate returned to pre-exposure levels (washout). A mean and standard error were calculated for each segment and significant responses to CGRP were determined by comparison to baseline firing rate using a standard T-test (Sigmaplot Software, SPSS Inc.; P ≤ 0.05).

To determine the location of each recorded neuron and characterize any regional selectivity in responses to CGRP, special attention was placed on determining the position of each electrode within the tissue slice. Electrodes were initially positioned within the tissue slices using stereoscopic visualization [7,29]. Once the recording of a neuron's firing rate was completed, the location of the electrode was visually confirmed. Using the ventral edge of the third ventricle as a reference point, the dorsal-ventral and lateral-medial coordinates were determined. The coronal position was estimated by preparing tissue slices with more tissue remaining on the left side of the third ventricle and recording the depth of the electrode from the surface of the tissue slice. Tissue slices were removed from the chamber, fixed in a 4% formalin solution overnight and prepared for additional sectioning by at least 2 hours in a 30% sucrose solution. Tissue slices were then re-sectioned to a thickness of 40 μm, mounted on gel coated slides, and the histological stain giemsa was used to identify hypothalamic nuclei to reconfirm the location of each recording.

## Abbreviations

CGRP: Calcitonin Gene Related Peptide; CNS: Central Nervous System; POAH: Preoptic and Anterior Regions of the Hypothalamus; PGE_2_: Prostaglandin E_2_; I_A_: Potassium A Current.

## Authors' contributions

DCB carried out the majority of cellular recordings and data analysis, with a significant amount of assistance from EMD and ERG. JDG conceived of the study and participated in its design, coordination and completion. All of the authors contributed to the drafting of this manuscript.
